# Supplementary material for: Recurrence of WHO-defined fast breathing pneumonia among infants, its occurrence and predictors in Pakistan: a nested case–control analysis
Source: BMJ Open. 2020 Jan 7;10(1):e035277. doi: 10.1136/bmjopen-2019-035277 (PMC6955570; doi:10.1136/bmjopen-2019-035277)
Supplement: Supplementary data [file bmjopen-2019-035277supp002.pdf]

Supplementary file

Recurrence by season

	Recurrent Fast breathing		
	Count	%	N
Jan	15	3%	445
Feb	16	6%	281
Mar	14	4%	328
Apr	11	3%	380
May	12	3%	343
Jun	16	5%	319
Jul	12	3%	380
Aug	14	5%	297
Sep	9	3%	263
Oct	13	3%	374
Nov	8	3%	272
Dec	11	3%	320

Selected vs all controls

		Potential controls	Selected controls	p-value
		N=2,455	N=302	
Arm	A	1,223 (49.8%)	149 (49.3%)	0.88
	B	1,232 (50.2%)	153 (50.7%)	
Child sex	Male	1,252 (51.0%)	167 (55.3%)	0.16
	Female	1,203 (49.0%)	135 (44.7%)	
Sibling number	<=3	1,731 (70.5%)	218 (72.2%)	0.55
	>3	724 (29.5%)	84 (27.8%)	
Room sharing number	<=4	1,398 (56.9%)	173 (57.3%)	0.91
	>4	1,057 (43.1%)	129 (42.7%)	
Housing Type	Katcha	138 (5.6%)	10 (3.3%)	0.013
	Mixed	218 (8.9%)	15 (5.0%)	
	Pakka	2,099 (85.5%)	277 (91.7%)	
Location of child school	Other	1,216 (55.0%)	153 (56.0%)	0.75
	Near factory/traffic fumes/public oven	993 (45.0%)	120 (44.0%)	
Drinking water	Improved drinking Water	2,218 (90.3%)	274 (90.7%)	0.83
	Unimproved drinking water	237 (9.7%)	28 (9.3%)	
Sanitation facilities	Improved sanitation facilities	2,227 (90.7%)	274 (90.7%)	0.99
	Unimproved sanitation facilities	228 (9.3%)	28 (9.3%)	
Cooking Fuel	Gas	2,098 (85.5%)	261 (86.4%)	0.65
	Wood/coal/animal dung	357 (14.5%)	41 (13.6%)	
Proper ventilation	Yes	2,209 (90.0%)	274 (90.7%)	0.68
	No	246 (10.0%)	28 (9.3%)	
Smoker in household	Yes	656 (26.7%)	70 (23.2%)	0.19
	No	1,799 (73.3%)	232 (76.8%)	
Pets with fur/feathers in household	Yes	535 (21.8%)	74 (24.5%)	0.28
	No	1,920 (78.2%)	228 (75.5%)	
Wheeze at presentstion	Yes	146 (5.9%)	23 (7.6%)	0.25
	No	2,309 (94.1%)	279 (92.4%)	
Weight-for-age z-score	Normal	1,414 (57.8%)	192 (63.6%)	0.053
	Under-weight	1,034 (42.2%)	110 (36.4%)	
Height-for-age z-score	Normal	1,323 (54.2%)	179 (59.9%)	0.065
	Stunting	1,116 (45.8%)	120 (40.1%)	
	Normal	1,969 (81.1%)	249 (83.0%)	0.42

Weight-for-height z-score	Wasting	459 (18.9%)	51 (17.0%)	
Age adequate vaccination	No	1,321 (53.8%)	168 (55.6%)	0.55
	Yes	1,134 (46.2%)	134 (44.4%)	
History of exclusive breastfeeding	Yes	1,563 (63.7%)	187 (61.9%)	0.55
	No	892 (36.3%)	115 (38.1%)	
