# Supplementary material for: Recurrence of WHO-defined fast breathing pneumonia among infants, its occurrence and predictors in Pakistan: a nested case–control analysis
Source: BMJ Open. 2020 Jan 7;10(1):e035277. doi: 10.1136/bmjopen-2019-035277 (PMC6955570; doi:10.1136/bmjopen-2019-035277)
Supplement: Author's manuscript [file bmjopen-2019-035277.draft_revisions.pdf]

BMJ Open is committed to open peer review. As part of this commitment we make the peer review history of every article we publish publicly available.

When an article is published we post the peer reviewers' comments and the authors' responses online. We also post the versions of the paper that were used during peer review. These are the versions that the peer review comments apply to.

The versions of the paper that follow are the versions that were submitted during the peer review process. They are not the versions of record or the final published versions. They should not be cited or distributed as the published version of this manuscript.

BMJ Open is an open access journal and the full, final, typeset and author-corrected version of record of the manuscript is available on our site with no access controls, subscription charges or pay-per-view fees (http://bmjopen.bmj.com).

If you have any questions on BMJ Open's open peer review process please email info.bmjopen@bmj.com

BMJ Open

Recurrence of World Health Organization defined Fast Breathing Pneumonia among Infants, its Occurrence and Predictors in Pakistan- A Nested Case Control Analysis.

Journal:	*BMJ Open*
Manuscript ID	bmjopen-2019-035277
Article Type:	Original research
Date Submitted by the Author:	30-Oct-2019

Complete List of Authors:	brown, nick; Uppsala Universitet, ; Aga Khan University,<br>Rizvi, Arjumand; Aga Khan Medical University, Pediatrics and Child Health<br>kerai, salima<br>Nisar, Muhammad; Aga Khan University, Pediatrics and Child Health<br>Rehman, Najeeb; Paediatrics and Child Health<br>Baloch, Benazir; Aga Khan University, Pediatrics and Child Health<br>Jehan, Fyezah; Aga Khan University, Paediatrics and Child Health; Fyezah Jehan,

Keywords:	EPIDEMIOLOGY, Paediatric infectious disease & immunisation < PAEDIATRICS, Tropical medicine < INFECTIOUS DISEASES

SCHOLARONE™
Manuscripts

Recurrence of World Health Organization defined Fast Breathing Pneumonia among Infants, its Occurrence and Predictors in Pakistan- A Nested Case Control Analysis.

Nick Brown (1,2) : nick.brown@kbh.uu.se

Arjumand Rizvi (2) arjumand. rizvi@aku.edu

Salima Kerai (3) salimakerai@gmail.com, skerai@mail.ubc.ca

Imran Nisar (2) imran.nisar@aku.edu

Najeeb Rehman (2) najeeb.rehman@aku.edu

Benazir Balouch (2) benazir.balouch@aku.edu

Fyezah Jehan (2) fyezah.jehan@aku.edu

Author affiliations

1. International Child and Maternal Health department, Institutionen för kvinnors och barns hälsa, Uppsala University, Uppsala, Sweden

2. Department of Pediatrics and Child Health, Aga Khan University, Karachi, Pakistan

3. University of British Columbia, Vancouver, Canada

Corresponding author

Nick Brown

International Child and Maternal Health department

Akademiska sjukhuset

Uppsala University

Uppsala

751 85

Sweden

Abstract: 240 words

Main text: 2,511words

For peer review only

Recurrence of World Health Organization defined Fast Breathing Pneumonia in children in Pakistan- A Nested Case Control Analysis.

Abstract

Objectives

Studies in Low- and Middle- Income Countries have shown an adverse association between environmental exposures including poverty. There is little literature from South Asia. We aimed to test the associations between housing, indoor air pollution and children's respiratory health and recurrent fast breathing pneumonia in a poor urban setting in Pakistan.

Setting

Primary health centres in a peri-urban slum in Karachi, Pakistan

Methods

Nested matched case control study within a non-inferiority randomized controlled trial of fast breathing pneumonia (RETAPP) in peri-urban slums of Karachi, Pakistan. Cases were children aged 2 to 60 months enrolled in RETAPP with fast breathing pneumonia who presented again with fast breathing between 8 weeks and 12 months after full recovery. Controls, selected in a 2:1 ratio, were age matched participants who did not re-present.

Multivariable conditional logistic regression analysis was undertaken to explore associations with potentially modifiable environmental predictors including housing type, indoor air quality, exposure to tobacco smoke, outdoor pollution, household crowding, water and sanitation quality, nutritional status, immunization completeness, breastfeeding and airways hyperactivity.

Results: Fast breathing recurred in 151 (3.7%) of children out of the total (4,003) enrolled in the trial. Poor quality housing of either katcha or mixed type strongly predicted recurrence with aMOR (95% CI) of 2.43 (1.02- 5,80) and 2.44 (1.11- 5.38) respectively. Poor air quality, cooking fuel, inadequate ventilation, nutritional status,

For peer review only - http://bmjopen.bmj.com/site/about/guidelines.xhtml

WASH index, wheeze at first presentation and group of initial trial assignment were not independently predictive of recurrence

Conclusion

Poor quality housing, a marker of poverty, independently predicted recurrence of pneumonia

Trial registration: Clinical Trials (NIH) Register NCT02372461

For peer review only

Strengths and limitations

Strengths

Little data from Asia in this area

Excellent follow up with complete baseline data

Limitations

Population homogeneity

No direct chemical measures of environmental exposures

Introduction

Child pneumonia contributes approximately 30% of the total global pneumonia mortality (1,2, 3,4, 5) but, despite a gradual recent fall in both incidence and case fatality rates over the Millennium Development Goal (MDG) era (1990-2015), it remains, by some distance, the single largest contributor to global post neonatal mortality (6,7) .

A recent review for the Child Health Epidemiology Action Group (CHERG) estimated an incidence of community–acquired childhood pneumonia in LMICs of 0.22 (IQR 0.11-0.51) episodes per child per year of which 11.5 % progress to severe episodes (8). Recent data estimate 120 million illness episodes per year of which between 880,000 and 935,000 cases are fatal. Pneumonia, therefore, contributes of the order of 15 % of the total 5.6 million annual under five year child deaths. The vast majority of the fatalities occur in Low- and Middle- Income Countries in settings where resources are more stretched and populations more susceptible and most in the first 2 years of life (6, 9). It is a disease of poverty, strongly related to household overcrowding with undernutrition increasing case fatality (10, 11)

Childhood pneumonia is intimately linked to poverty in all its manifestations (6). These include overcrowding, undernutrition and the use of indoor fossil fuels (10, 11). The relative contribution of each is hard to ascertain epidemiologically as each of the factors is so closely linked to the others but have all been shown to be independent predictors (6, 7,12-17)

Though there is some literature on environmental predictors of recurrent pneumonia, there is little to date from Asia (12,13). Given the prevalence of modifiable risk factors in peri urban Pakistan we aimed to establish the magnitude of their relative contributions in predicting recurrent episodes of WHO-defined fast breathing pneumonia. (18)

For peer review only - http://bmjopen.bmj.com/site/about/guidelines.xhtml

Methods

We undertook a nested case control study couched within the 'RETAPP* (Randomised Trial of Amoxicillin versus Placebo for Pneumonia) study (NCT02372461) (18,19). The methods and reporting (supplementary table 3) were based on STROBE guidance. In brief, RETAPP was a double blinded, randomized controlled non-inferiority trial undertaken between November 2014 and November 2017 in a poor peri urban slum area (Bin Qasim town) in Karachi, Pakistan for which the Aga Khan University has provided demographic surveillance for many years. It tested the hypothesis that treatment failure rates in children aged 2 to 59 months with WHO-defined fast breathing pneumonia without chest indrawing (15) or other signs would be non-inferior in children given a placebo to those treated according to WHO guidance with a 3 day course of amoxicillin prescribed according to weight bands and approximating 45 mg/ kg twice daily (WHO). A child met inclusion criteria if s/he presented with a respiratory rate of ≥ 50 breaths per minute (2-11 months) or ≥ 40 breaths per minute (12-59 months). A trained lady health worker and a physician assessed respiratory rate independently and a child was designated a fast-breather if there was agreement. Wheeze was assessed through auscultation by study physician. All children with wheeze received up to three doses of inhaled bronchodilator according to WHO-IMCI guidelines (18). Respiratory rate was reassessed after each inhalation. Only persistent fast breathers were retained. Children were excluded if there was associated lower chest indrawing, any danger signs, and use of antibiotics in last 48 hours, hospitalization in last two weeks, pedal edema, known tuberculosis, asthma or other severe illness. The primary outcome was composite treatment failure 3 days after randomization. A total of 4,003 children were included in the primary analysis.

Infant mortality in the area is 78/1000 live-births, one in six deaths attributable to pneumonia (DSS, 2012). The major source of income in the area is fishing and manual labor. Average household income is low with 70% of the adult population earning less than 5 USD/day. Household size typically ranges from 5-11 members/household and about 12% of the dwellers use solid fuels in the form of open fire stoves, and wood burners or animal dung for indoor energy. The remainder use closed stoves and natural gas. Due to the long standing presence of a

For peer review only - http://bmjopen.bmj.com/site/about/guidelines.xhtml

demographic surveillance by the Aga Khan University through the primary healthcare centers, the majority of children with any illness are brought back by the caregivers for follow-up and management of their child's subsequent illnesses. The trial and case control studies were based in 4 of the health centres.

Nested case control study: All previously randomized participants were eligible for inclusion in the nested case control study A case was defined as a child previously randomised who re-presented with a recurrent episode of isolated fast breathing between eight weeks to one year of the initial episode. Eight weeks was considered sufficient wash-out period for resolution of prior episode of fast breathing and the endpoint of a year was chosen for consistency with hospital studies of recurrent pneumonia (20).

For case selection, children enrolled in RETAPP, who presented again with a recurrent fast breathing episode, were identified through their DSS identification number, date of birth and first name. Children with missing or incorrectly recorded identification numbers or non-matching date of birth of birth or first names between first and second visit were excluded. Controls were defined as children between 2-59 months of age, enrolled in RETAPP trial and who did not present with a recurrent episode of fast breathing during that defined time period. As the incidence of pneumonia is greater in younger children and because vaccination completion is age dependent, we matched each case with two controls of the same number of completed years of age. For each case, two matched controls were identified.

Data collection

Exposure information for both cases and controls was obtained from their baseline visit (at the time of enrolment in RETAPP trial). Data was extracted for both cases and controls on several possible candidate predictors for fast breathing recurrence including: sex; sibling number; number of people reported to sleep in same room; exclusive breast feeding (up to six months of age); type of housing (based on material of roof, wall and floor); water and sanitation (WASH), status dichotomised as adequate or otherwise; nutrition status (based on WHO anthropometric z-scores of height for age and weight for age (a score of > - 2 SD being categorized as stunting and malnourished respectively); completeness of vaccination (dichotomised, yes/no)

For peer review only - http://bmjopen.bmj.com/site/about/guidelines.xhtml

defined according to the expanded program for immunization; the presence of wheeze at first presentation and the presence of pet animal in house. For completeness, we evaluated the association with the original group of randomization to assess any predictive or protective effect from earlier antibiotic treatment.

Sample size and statistical analysis: The primary outcome was the recurrence of a fast breathing episode between 2 and 12 months after the index (RETAPP randomized) episode diagnosed, as in the main trial, by two independent assessments at presentation

Sample size estimation was based on an Odds Ratio of 2.5 by the presence of adverse environmental exposure representing a public health risk using an alpha of 5% and power of 80% (beta 20 %). Based on pilot data of a 12% exposure prevalence of inadequate indoor air quality, in the population and a case control ratio of 1:2, the minimum estimated sample size required was 106 cases and 211 controls, a total of 317 children. We ultimately included all 151 children with records of recurrence and 302 controls to enhance the analysis.

All analyses were undertaken using STATA version 15 (Stata Corp, Texas). Data was summarized using frequencies and proportions. The predictors of recurrent fast breathing were determined by multivariable conditional logistic regression after initial univariable analysis. Variables significant at $p < 0.2$ in the univariable analysis were included in the fully adjusted model. The final model was constructed using backward elimination, variables being retained if $p < 0.1$. Results were presented as adjusted matched Odds Ratios (MOR). Plausible interaction and multi-collinearity was additionally assessed. A p value of > 0.05 was deemed statistically significant.

To allow for any intra-individual non-independent correlations between observations in children with more than one recurrence presentation, we additionally undertook an exploratory sensitivity analysis of intraclass correlation (ICC). This showed a very low ICC (variance=0.0001, ICC=0) suggesting that each case could be treated as an independent variable. The final analysis, therefore, used simple conditional logistic regression rather than a mixed effects model.

For peer review only - http://bmjopen.bmj.com/site/about/guidelines.xhtml

Ethical approval for the original trial was obtained both from the Aga Khan University (2786-pee-ERC-13). As the original trial was initially UK funded, we also obtained endorsement from the University of Southampton ethics' committee (ID 8700-December 2013)

The concept of the original trial was introduces to parents in the communities served by the health centres before recruitment began, but, there was no specific patient or public involvement in the design of the study

Results

Between November 2014 and November 2017, 53,838 children 2-59 months were self-referred to the primary health care center. Of these, 7,894 presented primarily with cough and tachypnea and, of these, 4,002 were eligible for and consented to enrolment in the RETAPP trial. Of these, 151 (3.7 %) met the criteria for eligibility in terms of a recurrence between eight weeks to one year of initial enrollment (cases) (figure 1). There was no clear seasonal pattern to incidence of recurrent pneumonia (supplementary file 1). We selected 302 children of the 2,757 who did not experience a recurrent episode (a 2:1 ratio) matched by age band. The selected controls were compared to the non-selected children without recurrence and shown to be similar in terms of baseline variables (supplementary file 1)

Univariable analysis (table 1) showed a significant predictive effect of poorer housing type and drinking water and protective effect of the presence of a household smoker and incomplete vaccination. In the multivariable analysis (table 2), only poor housing quality remained predictive: with aMORs (95% CI) of: katcha 2.43 (1.02- 5.80, p 0.04) and mixed 2.44 (1.11- 5.38, p 0.03). Household smoking and non-completion of vaccination remained protective. There was no predictive effect from: undernutrition; sex; inadequate ventilation; absence of breastfeeding; type of household fuel used; sibling number; bedroom crowding; wheeze at first presentation; household pet animals; sanitation or the proximity of the child's school to factories, traffic fumes or public ovens.

For peer review only - http://bmjopen.bmj.com/site/about/guidelines.xhtml

Table 1: baseline demographic variables in cases and controls

	Control	Case
	N=302	N=151
**Child sex**		
Male	167 (55.3%)	83 (55.0%)
Female	135 (44.7%)	68 (45.0%)
**Sibling number**		
<=3	218 (72.2%)	108 (71.5%)
>3	84 (27.8%)	43 (28.5%)
**Room sharing number**		
<=4	173 (57.3%)	81 (53.6%)
>4	129 (42.7%)	70 (46.4%)
**Housing Type**		
Katcha	10 (3.3%)	13 (8.6%)
Mixed	15 (5.0%)	16 (10.6%)
Pakka	277 (91.7%)	122 (80.8%)
**Used improved drinking water source**	274 (90.7%)	128 (84.8%)
**Used improved sanitation facilities**	274 (90.7%)	135 (89.4%)
**Cooking Fuel**		
Gas	261 (86.4%)	126 (83.4%)
Wood/coal/animal dung	41 (13.6%)	25 (16.6%)
**Location of child school**		
Otherwise	153 (56.0%)	70 (49.6%)
Near factory/traffic fumes/public oven	120 (44.0%)	71 (50.4%)
N	273	141
**Proper ventilation in house**	274 (90.7%)	140 (92.7%)
**Current smoker in household**	70 (23.2%)	22 (14.6%)
**Pets in household**	74 (24.5%)	32 (21.2%)
**Wheeze at presentation**	23 (7.6%)	12 (7.9%)
**Under-weight**	110 (36.4%)	60 (39.7%)
**Stunting**	120 (40.1%)	71 (47.3%)
N	299	150
**Wasting**	51 (17.0%)	29 (19.2%)
N	300	151
**Received age adequate vaccination**	134 (44.4%)	84 (55.6%)
**History of exclusive breastfeeding**	187 (61.9%)	93 (61.6%)

For peer review only - http://bmjopen.bmj.com/site/about/guidelines.xhtml

Table 2: Matched analysis showing effect of predictors on recurrent fast breathing

Predictors	Unadjusted		Adjusted	
	Matched OR (95% CI)	p-value	Matched OR (95% CI)	p-value
**Randomization group**				
Amoxicillin	1.29(0.87-1.91)	0.205	--	--
Placebo	Ref.		Ref.	
**Child sex**				
Male	Ref.		Ref.	
Female	1.01(0.69-1.50)	0.947	--	--
**Sibling number**				
<=3	Ref.		Ref.	
>3	1.03(0.67-1.59)	0.883	--	--
**Room sharing number**				
<=4	Ref.		Ref.	
>4	1.15(0.78-1.70)	0.469	--	--
**Housing Type**				
Katcha	2.80(1.22-6.44)	0.015	2.43(1.02-5.80)	0.045
Mixed	2.53(1.18-5.46)	0.018	2.44(1.11-5.38)	0.026
Pakka	Ref.		Ref.	
**Unimproved drinking water**	1.86(0.99-3.49)	0.053	1.72(0.89-3.36)	0.109
**Unimproved sanitation facilities**	1.16(0.61-2.21)	0.655	--	--
**Cooking Fuel**				
Gas	Ref.		Ref.	
Wood/coal/animal dung	1.28(0.73-2.22)	0.387	--	--
**Location of child school**				
Otherwise	Ref.			
Near factory/traffic fumes/public oven	1.28(0.84-1.95)	0.253	--	--
**No proper ventilation in house**	0.78(0.38-1.59)	0.487	--	--
**Current smoker in household**	0.57(0.34-0.96)	0.033	0.53(0.30-0.91)	0.022
**Pets in household**	0.83(0.52-1.33)	0.433	--	--
**Wheeze at presentation**	1.05(0.50-2.18)	0.901	--	--
**Underweight**	1.16(0.77-1.74)	0.486	--	--
**Stunted**	1.39(0.91-2.14)	0.129	--	--
**Wasted**	1.16(0.70-1.94)	0.566	--	--
**Age adequate vaccination**				
No	0.62(0.41-0.93)	0.021	0.61(0.40-0.94)	0.025
Yes	Ref.		Ref.	
**History of exclusive breastfeeding**				
Yes	Ref.		Ref.	
No	1.01(0.68-1.52)	0.946	--	--

For peer review only

For peer review only - http://bmjopen.bmj.com/site/about/guidelines.xhtml

For peer review only

For peer review only - http://bmjopen.bmj.com/site/about/guidelines.xhtml

Discussion

Our findings, to our knowledge are the first to show the burden of recurrence fast breathing pneumonia, 3.7 % of children re-presenting with a new pneumonia episode within a year of recovery from a previous episode.

Our starting premise was the association between environmental stressors and recurrent pneumonia. In our population, we showed no direct effect of indoor air pollution or water and sanitation exposures assessed by parent reported questionnaire. We did, however, show a significant association between housing quality, a proxy for poverty, and recurrence. A total of 8.3 % of the children lived in 'katcha' or mixed housing, forms of mud brick buildings often made as temporary homes for the poorest families which, even by urban slum standards, are rudimentary.

Childhood pneumonia is intimately linked to poverty in all its manifestations. These include overcrowding, undernutrition, incomplete vaccination and the use of indoor air pollution (11,12, 17). Sonego's meta-analysis estimated effect sizes for a range of socio-economic predictors and found that early pneumonia mortality was significantly related to young maternal age, low maternal education and low socio-economic status (12).

Poverty in all its manifestations is a crude, but sensitive, marker for adverse environmental stress (1-8, 10-14, 16). Not all poverty phenotypes are as easily measurable as, for example, the 'classic' markers: nutritional status; household size; completeness of vaccination; exposure to contaminated drinking water and airborne fine particulate matter. There is substantial evidence that one pathway is of chronic low grade inflammation demonstrable, for example by raised mean C reactive protein in more deprived populations (22) and of subtle micronutrient deficiencies and low level intergenerational psychological stress (23) all of which can be immune suppressant. Other factors increasing susceptibility include the mediating effects of low maternal education, maternal depression and impaired mother child interaction on childhood illness (24)

For peer review only

Recent Global Burden of Disease analysis indicated that poverty was the single largest contributor to childhood illness in Sub Saharan Africa (25) though any association between poverty, education, family size, sanitation and fuel use is complex. In recent work in Pakistan, Nasir showed multiple inter-related pathways (26). In Ram's study in Bangladesh (26) in children aged under 5 years with pneumonia where comparable proportions to our study lived in households using gas (85 % vs 86 % in Karachi) suggested that improved ventilation and the reduction of overcrowding were the single largest preventable determinants of illness. Tin roofs, low socio economic status and male sex were other predictors, but, as in our study particulate matter per se was not (27)

One explanation might be that our markers of exposure, a parental questionnaire at enrolment is insufficiently sensitive to discriminate between indoor air pollution and water safety and that direct measures of, for example, fine particles and coliform count respectively are required. Another factor might be that our population was too homogenous, all households being poor and that a degree of indoor air pollution exposure was the norm. Crude nutritional status by weight for age z score is also so prevalent (underweight 36 .4 % and stunted 40%) that any differences were lost.

The relative contribution of each airborne pollutant is arguably harder to ascertain epidemiologically as each of the factors, black carbon, fossil fuel, fine particulate matter (PM 2.5) and carbon monoxide (CO) , is so closely linked to all the others (7) Extrapolating this, Adaji's meta-analysis showed no association between home CO exposure and pneumonia. Fine particulate matter was only significant when using fuel as a proxy for exposure and not when measured directly highlighting the issues with standardizing exposure measurement in such settings (16)

It would be in keeping with this hypothesis that household size is too blunt a tool, the majority of the families consisting of 10 or more members and multiple room sharing inevitable. Incomplete vaccination status similarly was so common (55.6 %) that interpretation of the result difficult and likely to be explained by confounding. In addition, most episodes of fast breathing pneumonia are known to be viral against which the common capsulated polysaccharide antigens are unlikely to be protective.

For peer review only - http://bmjopen.bmj.com/site/about/guidelines.xhtml

Given the association of poverty with recurrent pneumonia and the cost of cigarettes it seems plausible that the effect of household smoking is confounded by wealth. To corroborate this hypothesis, we have showed an association between lower numbers of smokers and household wealth.

There are several strengths of the study. It was undertaken in a stable population within a well-established demographic surveillance system and a very high rate of capture of episodes

There are, though, some limitations. In a few cases where there was lack of database linkage due to incorrectly recorded demographic surveillance identity. Of the total 4,002 RETAPP enrolled children, 710 (17.7%) had no DSS identifier It is also possible that a small proportion of recurrent cases may have presented to other facilities (private or out of the city) which may have led to an underestimate of incidence of recurrence. We did not directly measure particulate matter density to quantify solid fuel exposure and our questionnaire might have been too insensitive to detect differences. Finally, our population may have been too homogenous to detect small differences in exposure effect.

Conclusion

Recurrent fast breathing pneumonia in this deprived peri urban population in Pakistan was independently predicted by poverty as manifested by poor housing quality.

Acknowledgements

We would like to acknowledge patients and families, the RETAPP study team, including community health workers, demographic surveillance workers and the committed physicians who took care of patients.

For peer review only - http://bmjopen.bmj.com/site/about/guidelines.xhtml

Funding

This work is jointly funded by the MRC-Wellcome-DFID through the Joint Global Health Trials, [grant number MR/L004283/1] and the Bill and Melinda Gates Foundation [grant number OPP1158281].

Fyezah Jehan and Imran Nisar received funding from the National Institute of Health's Fogarty International Center [Grant Number 1 D43 TW007585-01].

Competing interests

The authors declare no competing interests.

Authors' contributions

FJ conceived the idea; FJ, SK, IN, BB and NB designed the study. FJ, IN, BB, NB, NR were involved in conduct of the study. NB, AR, FJ, SK, IN, NB and NR were involved in statistical analysis. FJ, NB and SK wrote the first draft of the manuscript and NB rewrote new drafts based on input from co-authors IN, NB, BB and NR. All authors read and approved the final manuscript.

Ethics

The RETAPP study received ethical approval by the Ethics Review Committee of Aga Khan University (ERC reference number 2786-Ped-ERC-13).

Data sharing

Data will available on public domain 2 years after the completion of RETAPP trial and publication of the main manuscripts. Processes will be developed to facilitate data sharing for scientific utilization in a collaborative manner.

For peer review only

References

1. Boloursaz MR, Lotfian F, Aghahosseini F, Cheraghvandi A, Khalilzadeh S, Farjah A, et al. Epidemiology of Lower Respiratory Tract Infections in Children. J Compr Ped. 2013;3(3):93–8.

2. Levels and Trends in Child Mortality: Report 2015: Estimates Developed by the UN Inter-Agency Group for Child Mortality Estimation (IGME). World Health Organization: New York: United Nations Children's Fund.

3. Walker CLF, Rudan I, Liu L, et al. Global burden of childhood pneumonia and diarrhoea. The Lancet 2013;**381**(9875):1405-16.

4. Liu L, Oza S, Hogan D, et al. Global, regional, and national causes of under-5 mortality in 2000–15: an updated systematic analysis with implications for the Sustainable Development Goals. The Lancet 2016;**388**(10063):3027-35.

5. UNICEF 2018. https://data.unicef.org/topic/child-health/pneumonia/. Last accessed 5/10/19

6. Leung, Christi et al (2016). Prevention and Control of Childhood Pneumonia and Diarrhea. Pediatr Clin North Am. ; 63(1): 67–79. doi:10.1016/j.pcl.2015.08.003

‘

7. Rudan I, O'Brien KL et al 2013. Epidemiology and etiology of childhood pneumonia in 2010: estimates of incidence, severe morbidity, mortality, underlying risk factors and causative pathogens for 192 countries. J Glob Health. 2013;3:010401. Medline:23826505

For peer review only

8. Zhang S, Sammon P. Cost of management of severe pneumonia in young children: systematic analysis. Journal of Global Health 2016. doi: 10.7189/jogh.06.010408

9. Tuti T, Agweyu A, Mwaniki P, Peek N, English M, on behalf of the Clinical Information Network Author Group. An exploration of mortality risk factors in non-severe pneumonia in children using clinical data from Kenya. BMC Medicine. 2017 Nov 13; 15: 201

10. Scott JA, Brooks WA et al. Pneumonia research to reduce childhood mortality in the developing world. J Clin Invest. 2008;118(4):1291-1300. https://doi.org/10.1172/JCI33947

11. Niessen L, ten Hove A. Comparative impact assessment of child pneumonia interventions. Bull World Health Organ 2009;87:472–480 | doi:10.2471/BLT.08.050872

12. Sonego M, Pellegrin MC, Becker G, Lazzerini M (2015) Risk Factors for Mortality from Acute Lower Respiratory Infections (ALRI) in Children under Five Years of Age in Low and Middle-Income Countries: A Systematic Review and Meta-Analysis of Observational Studies. PLOS ONE 10(1): e0116380.https://doi.org/10.1371/journal.pone.0116380

13. Adaji EE, Phaley R, Clifford M. Understanding the effect of indoor air pollution on pneumonia in children under 5 in low- and middle-income countries: a systematic review of evidence. Environ Sci Pollut Res Int. 2019; 26(4): 3208–3225. doi: 10.1007/s11356-018-3769-1

For peer review only

14. Bradley RH, Putnick DL. Housing Quality and Access to Material and Learning Resources Within the Home Environment in Developing Countries. Child Dev. 2012 Jan; 83(1): 76–91. doi: 10.1111/j.1467-8624.2011.01674.x

15. Adaji EE, Ekezie W, Clifford M, Phalkey R. Understanding the effect of indoor air pollution on pneumonia in children under 5 in low- and middle-income countries: a systematic review of evidence. Environmental Science and Pollution Research (2019) 26:3208–3225 https://doi.org/10.1007/s11356-018-3769-1

16. Bouazza N, Foissac F et al. Fine particulate pollution and asthma exacerbations. Arch Dis Child 2017;0:1–4. doi:10.1136/archdischild-2017-312826

17. Been JV, Sheikh A. Tobacco control policies in relation to child health and perinatal health outcomes. Arch Dis Child 2018;103:817–819.

18. World Health Organisation. https://www.ncbi.nlm.nih.gov/books/NBK264164/

19. Jehan F, Nisar MI, Kerai S, et al. A double blind community-based randomized trial of amoxicillin versus placebo for fast breathing pneumonia in children aged 2-59 months in Karachi, Pakistan (RETAPP). BMC infectious diseases 2016;**16**(1):1.

20. Jehan F, Nisar I, Kerai S, **Brown N**, Ambler G, Zaidi A. Should fast breathing pneumonia cases be treated with antibiotics? The scientific rationale for revisiting management in Low and Middle Income Countries International Journal of Infectious Diseases. International Journal of Infectious Diseases 85 (2019) 64–66. https://doi.org/10.1016/j.ijid.2019.05.035

For peer review only

21. Izadnegahdar R, Fox MP, Thea DM, et al. Frequency and trajectory of abnormalities in respiratory rate, temperature and oxygen saturation in severe pneumonia in children. The Pediatric infectious disease journal 2012;**31**(8):863.

22. Schmeer KK, Yoon A. Socioeconomic status inequalities in low-grade inflammation during childhood. Archives of Disease in Childhood 2016;**101:**1043-1047.

23. Burris HH, Lorch SA, Kirpalani H, et al
Racial disparities in preterm birth in USA: a biosensor of physical and social environmental exposures. Archives of Disease in Childhood 2019;**104:**931-935.

24. Brown N, Finch Jenna, Jelena Obradovic, Aisha Yousafzai. Maternal Care mediates the effects of nutrition and responsive stimulation interventions on young children's growth. Child: health, care and development. DOI:10.1111/cch.12466

25. Lim SS, Vos T, Flaxman AD, et al. A comparative risk assessment of burden of disease and injury attributable to 67 risk factors and risk factor clusters in 21 regions, 1990–2010: a systematic analysis for the Global Burden of Disease Study 2010. The lancet 2013;**380**(9859):2224-60.

26. Nasir ZA, Murtaza F, Colbeck I. Role of poverty in fuel choice and exposure to indoor air pollution in Pakistan. Journal of Integrative Environmental Sciences 2015;**12**(2):107-17.

27. Ram PK, Dutt D, Silk BJ, et al. Household air quality risk factors associated with childhood pneumonia in urban dhaka, bangladesh. The American journal of tropical medicine and hygiene 2014;**90**(5):968-75.

For peer review only

For peer review only

For peer review only - http://bmjopen.bmj.com/site/about/guidelines.xhtml

For peer review only

For peer review only - http://bmjopen.bmj.com/site/about/guidelines.xhtml

For peer review only

For peer review only - http://bmjopen.bmj.com/site/about/guidelines.xhtml

Figure I: flow chart

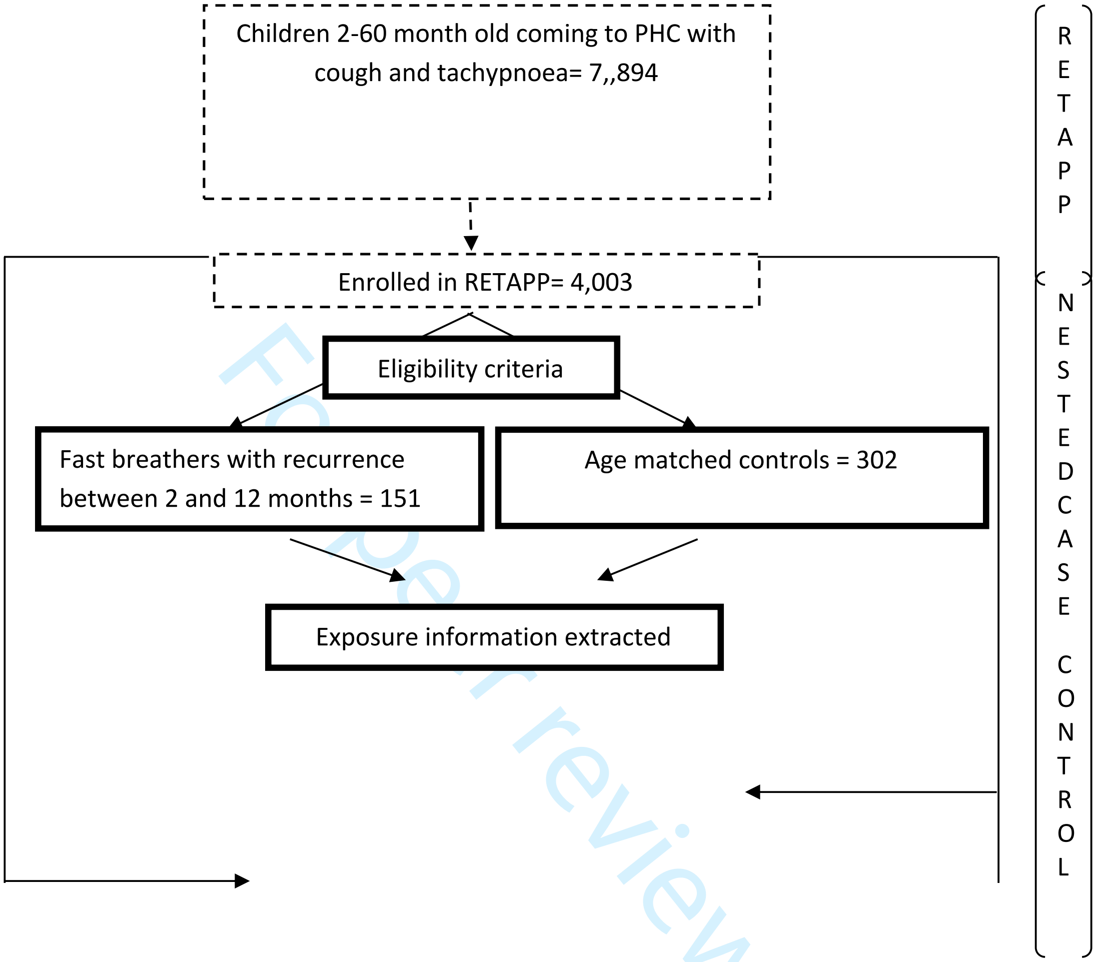

For peer review only

Supplementary file

Recurrence by season

	Recurrent Fast breathing		
	Count	%	N
Jan	15	3%	445
Feb	16	6%	281
Mar	14	4%	328
Apr	11	3%	380
May	12	3%	343
Jun	16	5%	319
Jul	12	3%	380
Aug	14	5%	297
Sep	9	3%	263
Oct	13	3%	374
Nov	8	3%	272
Dec	11	3%	320

For peer review only

Selected vs all controls

		Potential controls N=2,455	Selected controls N=302	p-value
Arm	A	1,223 (49.8%)	149 (49.3%)	0.88
	B	1,232 (50.2%)	153 (50.7%)	
Child sex	Male	1,252 (51.0%)	167 (55.3%)	0.16
	Female	1,203 (49.0%)	135 (44.7%)	
Sibling number	<=3	1,731 (70.5%)	218 (72.2%)	0.55
	>3	724 (29.5%)	84 (27.8%)	
Room sharing number	<=4	1,398 (56.9%)	173 (57.3%)	0.91
	>4	1,057 (43.1%)	129 (42.7%)	
Housing Type	Katcha	138 (5.6%)	10 (3.3%)	0.013
	Mixed	218 (8.9%)	15 (5.0%)	
	Pakka	2,099 (85.5%)	277 (91.7%)	
Location of child school	Other	1,216 (55.0%)	153 (56.0%)	0.75
	Near factory/traffic fumes/public oven	993 (45.0%)	120 (44.0%)	
Drinking water	Improved drinking Water	2,218 (90.3%)	274 (90.7%)	0.83
	Unimproved drinking water	237 (9.7%)	28 (9.3%)	
Sanitation facilities	Improved sanitation facilities	2,227 (90.7%)	274 (90.7%)	0.99
	Unimproved sanitation facilities	228 (9.3%)	28 (9.3%)	
Cooking Fuel	Gas	2,098 (85.5%)	261 (86.4%)	0.65
	Wood/coal/animal dung	357 (14.5%)	41 (13.6%)	
Proper ventilation	Yes	2,209 (90.0%)	274 (90.7%)	0.68
	No	246 (10.0%)	28 (9.3%)	
Smoker in household	Yes	656 (26.7%)	70 (23.2%)	0.19
	No	1,799 (73.3%)	232 (76.8%)	
Pets with fur/feathers in household	Yes	535 (21.8%)	74 (24.5%)	0.28
	No	1,920 (78.2%)	228 (75.5%)	
Wheeze at presentstion	Yes	146 (5.9%)	23 (7.6%)	0.25
	No	2,309 (94.1%)	279 (92.4%)	
Weight-for-age z-score	Normal	1,414 (57.8%)	192 (63.6%)	0.053
	Under-weight	1,034 (42.2%)	110 (36.4%)	
Height-for-age z-score	Normal	1,323 (54.2%)	179 (59.9%)	0.065
	Stunting	1,116 (45.8%)	120 (40.1%)	
	Normal	1,969 (81.1%)	249 (83.0%)	0.42

For peer review only

Weight-for-height z-score	Wasting	459 (18.9%)	51 (17.0%)	
Age adequate vaccination	No	1,321 (53.8%)	168 (55.6%)	0.55
	Yes	1,134 (46.2%)	134 (44.4%)	
History of exclusive breastfeeding	Yes	1,563 (63.7%)	187 (61.9%)	0.55
	No	892 (36.3%)	115 (38.1%)	

For peer review only

STROBE Statement—checklist of items that should be included in reports of observational studies

	Item No	Recommendation	Page No
**Title and abstract**	1	(*a*) Indicate the study's design with a commonly used term in the title or the abstract	Yes
		(*b*) Provide in the abstract an informative and balanced summary of what was done and what was found	Yes
**Introduction**			
Background/rationale	2	Explain the scientific background and rationale for the investigation being reported	Yes 2-3
Objectives	3	State specific objectives, including any prespecified hypotheses	2-3
**Methods**			
Study design	4	Present key elements of study design early in the paper	5-7
Setting	5	Describe the setting, locations, and relevant dates, including periods of recruitment, exposure, follow-up, and data collection	5-7
Participants	6	(*a*) *Cohort study*—Give the eligibility criteria, and the sources and methods of selection of participants. Describe methods of follow-up<br>*Case-control study*—Give the eligibility criteria, and the sources and methods of case ascertainment and control selection. Give the rationale for the choice of cases and controls<br>*Cross-sectional study*—Give the eligibility criteria, and the sources and methods of selection of participants	5-7
		(*b*) *Cohort study*—For matched studies, give matching criteria and number of exposed and unexposed<br>*Case-control study*—For matched studies, give matching criteria and the number of controls per case	
Variables	7	Clearly define all outcomes, exposures, predictors, potential confounders, and effect modifiers. Give diagnostic criteria, if applicable	5-7
Data sources/ measurement	8*	For each variable of interest, give sources of data and details of methods of assessment (measurement). Describe comparability of assessment methods if there is more than one group	*5-7*
Bias	9	Describe any efforts to address potential sources of bias	5-7
Study size	10	Explain how the study size was arrived at	5-7
Quantitative variables	11	Explain how quantitative variables were handled in the analyses. If applicable, describe which groupings were chosen and why	5-7
Statistical methods	12	(*a*) Describe all statistical methods, including those used to control for confounding	5-7
		(*b*) Describe any methods used to examine subgroups and interactions	5-7
		(*c*) Explain how missing data were addressed	5-7
		(*d*) *Cohort study*—If applicable, explain how loss to follow-up was addressed<br>*Case-control study*—If applicable, explain how matching of cases and controls was addressed<br>*Cross-sectional study*—If applicable, describe analytical methods taking account of sampling strategy	5-7
		(*e*) Describe any sensitivity analyses	5-7

Continued on next page

For peer review only - http://bmjopen.bmj.com/site/about/guidelines.xhtml

Results			
Participants	13*	(a) Report numbers of individuals at each stage of study—eg numbers potentially eligible, examined for eligibility, confirmed eligible, included in the study, completing follow-up, and analysed	8+figure
		(b) Give reasons for non-participation at each stage	8
		(c) Consider use of a flow diagram	figure
Descriptive data	14*	(a) Give characteristics of study participants (eg demographic, clinical, social) and information on exposures and potential confounders	8+table 1
		(b) Indicate number of participants with missing data for each variable of interest	8
		(c) *Cohort study*—Summarise follow-up time (eg, average and total amount)	
Outcome data	15*	*Cohort study*—Report numbers of outcome events or summary measures over time	
		Case-control study—Report numbers in each exposure category, or summary measures of exposure	*8*
		Cross-sectional study—Report numbers of outcome events or summary measures	
Main results	16	(*a*) Give unadjusted estimates and, if applicable, confounder-adjusted estimates and their precision (eg, 95% confidence interval). Make clear which confounders were adjusted for and why they were included	8+table 2
		(*b*) Report category boundaries when continuous variables were categorized	8+table 2
		(*c*) If relevant, consider translating estimates of relative risk into absolute risk for a meaningful time period	-
Other analyses	17	Report other analyses done—eg analyses of subgroups and interactions, and sensitivity analyses	7
**Discussion**			
Key results	18	Summarise key results with reference to study objectives	13-15
Limitations	19	Discuss limitations of the study, taking into account sources of potential bias or imprecision. Discuss both direction and magnitude of any potential bias	13-15
Interpretation	20	Give a cautious overall interpretation of results considering objectives, limitations, multiplicity of analyses, results from similar studies, and other relevant evidence	13-15
Generalisability	21	Discuss the generalisability (external validity) of the study results	13-15
**Other information**			
Funding	22	Give the source of funding and the role of the funders for the present study and, if applicable, for the original study on which the present article is based	15

*Give information separately for cases and controls in case-control studies and, if applicable, for exposed and unexposed groups in cohort and cross-sectional studies.

Note: An Explanation and Elaboration article discusses each checklist item and gives methodological background and published examples of transparent reporting. The STROBE checklist is best used in conjunction with this article (freely available on the Web sites of PLoS Medicine at http://www.plosmedicine.org/, Annals of Internal Medicine at http://www.annals.org/, and Epidemiology at http://www.epidem.com/). Information on the STROBE Initiative is available at www.strobe-statement.org.

For peer review only - http://bmjopen.bmj.com/site/about/guidelines.xhtml

BMJ Open

Recurrence of World Health Organization defined Fast Breathing Pneumonia among Infants, its Occurrence and Predictors in Pakistan- A Nested Case Control Analysis.

Journal:	*BMJ Open*
Manuscript ID	bmjopen-2019-035277.R1
Article Type:	Original research
Date Submitted by the Author:	27-Nov-2019

Complete List of Authors:	brown, nick; Uppsala Universitet, ; Aga Khan University,<br>Rizvi, Arjumand; Aga Khan Medical University, Pediatrics and Child Health<br>kerai, salima<br>Nisar, Muhammad; Aga Khan University, Pediatrics and Child Health<br>Rahman, Najeeb; Aga Khan University, Paediatrics and Child Health<br>Baloch, Benazir; Aga Khan University, Pediatrics and Child Health<br>Jehan, Fyezah; Aga Khan University, Paediatrics and Child Health; Fyezah Jehan,

**Primary Subject Heading**:	Epidemiology
Secondary Subject Heading:	Communication
Keywords:	EPIDEMIOLOGY, Paediatric infectious disease & immunisation < PAEDIATRICS, Tropical medicine < INFECTIOUS DISEASES, Paediatric A&E and ambulatory care < PAEDIATRICS, Public health < INFECTIOUS DISEASES, Infection control < INFECTIOUS DISEASES

SCHOLARONE™
Manuscripts

Recurrence of World Health Organization defined Fast Breathing Pneumonia among Infants, its Occurrence and Predictors in Pakistan- A Nested Case Control Analysis.

Nick Brown (1,2) : nick.brown@kbh.uu.se
Orcid https://orcid.org/0000-0003-1789-0436

Arjumand Rizvi (2) arjumand.rizvi@aku.edu

Salima Kerai (3) salimakerai@gmail.com, skerai@mail.ubc.ca

Imran Nisar (2) imran.nisar@aku.edu

Najeeb Rahman (2) najeeb.rahman@aku.edu

Benazir Balouch (2) benazir.balouch@aku.edu

Fyezah Jehan (2) fyezah.jehan@aku.edu

Author affiliations

1. International Child and Maternal Health department, Institutionen för kvinnors och barns hälsa, Uppsala University, Uppsala, Sweden

2. Department of Pediatrics and Child Health, Aga Khan University, Karachi, Pakistan

3. University of British Columbia, Vancouver, Canada

Corresponding author

Nick Brown

International Child and Maternal Health department
Akademiska sjukhuset
Uppsala University
Uppsala
751 85
Sweden
Abstract: 261 words. Main text: 2,829 words

For peer review only

Recurrence of World Health Organization defined Fast Breathing Pneumonia in children in Pakistan- A Nested Case Control Analysis.

Abstract

Objectives

Studies in Low- and Middle- Income Countries have shown an adverse association between environmental exposures including poverty. There is little literature from South Asia. We aimed to test the associations between housing, indoor air pollution and children's respiratory health and recurrent fast breathing pneumonia in a poor urban setting in Pakistan.

Setting

Primary health centres in a peri-urban slum in Karachi, Pakistan

Methods

Nested matched case control study within a non-inferiority randomized controlled trial of fast breathing pneumonia (RETAPP) in peri-urban slums of Karachi, Pakistan. Cases were children aged 2 to 60 months enrolled in RETAPP with fast breathing pneumonia who presented again with fast breathing between 8 weeks and 12 months after full recovery. Controls, selected in a 2:1 ratio, were age matched participants who did not re-present.

Multivariable conditional logistic regression analysis was undertaken to explore associations with potentially modifiable environmental predictors including housing type, indoor air quality, exposure to tobacco smoke, outdoor pollution, household crowding, water and sanitation quality, nutritional status, immunization completeness, breastfeeding and airways hyperactivity.

Results: Fast breathing recurred in 151 (3.7%) of children out of the total (4,003) enrolled in the trial. Poor quality housing of either katcha or mixed type strongly predicted recurrence with adjusted Matched Odds Ratios (aMOR) of 2.43 (95 % CI 1.02- 5,80) and 2.44 (1.11- 5.38) respectively. Poor air quality, cooking fuel, inadequate ventilation, nutritional status, water,

For peer review only - http://bmjopen.bmj.com/site/about/guidelines.xhtml

sanitation and hygiene (WASH) index, wheeze at first presentation and group of initial trial assignment were not independently predictive of recurrence

Conclusion

Poor quality housing independently predicted recurrence of fast breathing pneumonia

Trial registration: Clinical Trials (NIH) Register NCT02372461

Strengths

Though there is some work from Low and Middle Income Countries related to environment and respiratory health, to date there has been little data from Asia

As a result of the existing infrastructure in the population studied, follow up and capture of recurrence of pneumonia was excellent

Matched design removes potential confounding effects of age and immunization status

Limitations

The population is relatively homogenous in terms of socio-economic status which may have blunted estimated effect sizes of some potential exposures

Though we used validated proxies for environmental exposure, these were not measured directly

For peer review only

Introduction

Child pneumonia contributes approximately 30% of the total global pneumonia mortality (1,2, 3,4, 5) but, despite a gradual recent fall in both incidence and case fatality rates over the Millennium Development Goal (MDG) era (1990-2015), it remains, by some distance, the single largest contributor to global post neonatal mortality (6,7) .

A recent review for the Child Health Epidemiology Action Group (CHERG) estimated an incidence of community–acquired childhood pneumonia in LMICs of 0.22 (IQR 0.11-0.51) episodes per child per year of which 11.5 % progress to severe episodes (8). Recent data estimate 120 million illness episodes per year of which between 880,000 and 935,000 cases are fatal. Pneumonia, therefore, contributes of the order of 15 % of the total 5.6 million annual under five year child deaths. The vast majority of the fatalities occur in Low- and Middle-Income Countries (LMICs) in settings where resources are more stretched and populations more susceptible and most in the first 2 years of life (6, 9). It is a disease of poverty, strongly related to household overcrowding with undernutrition increasing case fatality (10, 11)

Childhood pneumonia is intimately linked to poverty in all its manifestations (6). These include overcrowding, undernutrition and the use of indoor fossil fuels (10, 11). The relative contribution of each is hard to ascertain epidemiologically as each of the factors is so closely linked to the others but have all been shown to be independent predictors (6, 7,12-17)

Though there is some literature on environmental predictors of recurrent pneumonia, there is little to date from Asia (12,13). Given the prevalence of modifiable risk factors in peri urban Pakistan we aimed to establish the magnitude of their relative contributions in predicting recurrent episodes of World Health Organisation (WHO) defined fast breathing pneumonia. (18)

For peer review only

Methods

We undertook a nested case control study couched within the 'RETAPP* (Randomised Trial of Amoxicillin versus Placebo for Pneumonia) study (NCT02372461) (18,19). The methods and reporting were based on STROBE guidance (supplementary file 1). In brief, RETAPP was a double blinded, randomized controlled non-inferiority trial undertaken between November 2014 and November 2017 in a poor peri urban slum area (Bin Qasim town) in Karachi, Pakistan for which the Aga Khan University has provided demographic surveillance for many years. It tested the hypothesis that treatment failure rates in children aged 2 to 59 months with WHO-defined fast breathing pneumonia without chest indrawing (15) or other signs would be non-inferior in children given a placebo to those treated according to WHO guidance with a 3 day course of amoxicillin prescribed according to weight bands and approximating 45 mg/ kg twice daily (WHO). A child met inclusion criteria if s/he presented with a respiratory rate of ≥ 50 breaths per minute (2-11 months) or ≥ 40 breaths per minute (12-59 months) with or without fever. A trained lady health worker and a physician assessed respiratory rate independently and a child was designated a fast-breather if there was agreement. Wheeze was assessed through auscultation by study physician. All children with wheeze received up to three doses of inhaled bronchodilator according to WHO-IMCI guidelines (18). Respiratory rate was reassessed after each inhalation. Only persistent fast breathers were retained. Children were excluded if there was associated lower chest indrawing, any danger signs, and use of antibiotics in last 48 hours, hospitalization in last two weeks, pedal edema, known tuberculosis, asthma or other severe illness. The primary outcome was composite treatment failure 3 days after randomization. A total of 4,003 children were included in the primary analysis.

Infant mortality in the area is 78/1000 live-births, one in six deaths attributable to pneumonia (DSS, 2012). The major source of income in the area is fishing and manual labor. Average household income is low with 70% of the adult population earning less than 5 USD/day. Household size typically ranges from 5-11 members/household and about 12% of the dwellers use solid fuels in the form of open fire stoves, and wood burners or animal dung for indoor energy. The remainder use closed stoves and natural gas. Due to the long standing presence of a demographic surveillance by the Aga Khan University through the primary healthcare centers, the majority of children with any illness are brought back by the caregivers for

For peer review only - http://bmjopen.bmj.com/site/about/guidelines.xhtml

follow-up and management of their child's subsequent illnesses. The trial and case control studies were based in 4 of the health centres.

Nested case control study: All previously randomized participants were eligible for inclusion in the nested case control study A case was defined as a child previously randomised who re-presented with a recurrent episode of isolated fast breathing between eight weeks to one year of the initial episode. Eight weeks was considered sufficient wash-out period for resolution of prior episode of fast breathing and the endpoint of a year was chosen for consistency with hospital studies of recurrent pneumonia (19, 20, 21).

For case selection, children enrolled in RETAPP, who presented again with a recurrent fast breathing episode, were identified through their DSS identification number, date of birth and first name. Children with missing or incorrectly recorded identification numbers or non-matching date of birth of birth or first names between first and second visit were excluded. Controls were defined as children between 2-59 months of age, enrolled in RETAPP trial and who did not present with a recurrent episode of fast breathing during that defined time period. As the incidence of pneumonia is greater in younger children and because vaccination completion is age dependent, we matched each case with two controls of the same number of completed years of age. For each case, two matched controls were identified.

Data collection

Exposure information for both cases and controls was obtained from their baseline visit (at the time of enrolment in RETAPP trial). Data was extracted for both cases and controls on several possible candidate predictors for fast breathing recurrence including: sex; sibling number; number of people reported to sleep in same room; exclusive breast feeding (up to six months of age); type of housing (katcha- made of mud, straw, wood and dry leaves, pakka- rooves, walls and floors made of high quality material and mixed) ; water and sanitation (WASH), status dichotomised as adequate or otherwise; nutrition status (based on WHO anthropometric z-scores of height for age and weight for age (a score of > - 2 SD being categorized as stunting and malnourished respectively); completeness of vaccination (dichotomised, yes/no) defined according to the expanded program for immunization; the presence of wheeze at first presentation and the presence of pet animal in house. For completeness, we evaluated the association with the original group of randomization to assess any predictive or protective effect from earlier antibiotic treatment.

For peer review only - http://bmjopen.bmj.com/site/about/guidelines.xhtml

Sample size and statistical analysis: The primary outcome was the recurrence of a fast breathing episode between 2 and 12 months after the index (RETAPP randomized) episode diagnosed, as in the main trial, by two independent assessments at presentation

Sample size estimation was based on an Odds Ratio of 2.5 by the presence of adverse environmental exposure representing a public health risk using an alpha of 5% and power of 80% (beta 20 %). Based on pilot data of a 12% exposure prevalence of inadequate indoor air quality, in the population and a case control ratio of 1:2, the minimum estimated sample size required was 106 cases and 211 controls, a total of 317 children. We ultimately included all 151 children with records of recurrence and 302 controls to enhance the precision of the estimated effect size.

All analyses were undertaken using STATA version 15 (Stata Corp, Texas). Data was summarized using frequencies and proportions. The predictors of recurrent fast breathing were determined by multivariable conditional logistic regression after initial univariable analysis. Variables significant at $p < 0.2$ in the univariable analysis were included in the fully adjusted model. The final model was constructed using backward elimination, variables being retained if $p < 0.1$. Results were presented as adjusted matched Odds Ratios (aMOR). Plausible interaction and multi-collinearity was additionally assessed. A p value of > 0.05 was deemed statistically significant.

To allow for any intra-individual non-independent correlations between observations in children with more than one recurrence presentation, we additionally undertook an exploratory sensitivity analysis of intraclass correlation (ICC). This showed a very low ICC (variance=0.0001, ICC=0) suggesting that each case could be treated as an independent variable. The final analysis, therefore, used simple conditional logistic regression rather than a mixed effects model.

Ethical approval for the original trial was obtained both from the Aga Khan University (2786-pee-ERC-13). As the original trial was initially UK funded, we also obtained endorsement from the University of Southampton ethics' committee (ID 8700- December 2013)

For peer review only - http://bmjopen.bmj.com/site/about/guidelines.xhtml

Patient and public involvement

The concept of the original trial was introduced to parents in the communities served by the health centres before recruitment began, but, there was no specific patient or public involvement in the design of the study.

Results

Between November 2014 and November 2017, 53,838 children 2-59 months were self-referred to the primary health care center. Of these, 7,894 presented primarily with cough and tachypnea and, of these, 4,002 were eligible for and consented to enrolment in the RETAPP trial. Of these, 151 (3.7 %) met the criteria for eligibility in terms of a recurrence between eight weeks to one year of initial enrolment (cases) (figure 1). There was no clear seasonal pattern to incidence of recurrent pneumonia (supplementary file 2). We selected 302 children of the 2,757 who did not experience a recurrent episode (a 2:1 ratio) matched by age band. The selected controls were compared to the non-selected children without recurrence and shown to be similar in terms of baseline variables (supplementary file 2)

Univariable analysis (table 1) showed a significant association with poorer housing type and drinking water and protective effect of the presence of a household smoker and incomplete vaccination. In the multivariable analysis (table 2), only poor housing quality remained predictive: with aMORs (95% CI) relative to the pukka housing reference of: katcha 2.43 (1.02- 5.80, p 0.04) and mixed 2.44 (1.11- 5.38, p 0.03) respectively. There was no association with: undernutrition; sex; inadequate ventilation; absence of breastfeeding; type of household fuel used; sibling number; bedroom crowding; wheeze at first presentation; household pet animals; sanitation or the proximity of the child's school to factories, traffic fumes or public ovens.

For peer review only

Table 1: baseline demographic variables in cases and controls

	Control	Case
	**N=302**	**N=151**
**Child sex**		
Male	167 (55.3%)	83 (55.0%)
Female	135 (44.7%)	68 (45.0%)
**Sibling number**		
<=3	218 (72.2%)	108 (71.5%)
>3	84 (27.8%)	43 (28.5%)
**Room sharing number**		
<=4	173 (57.3%)	81 (53.6%)
>4	129 (42.7%)	70 (46.4%)
**Housing Type**		
Katcha	10 (3.3%)	13 (8.6%)
Mixed	15 (5.0%)	16 (10.6%)
Pakka	277 (91.7%)	122 (80.8%)
**Used improved drinking water source**	274 (90.7%)	128 (84.8%)
**Used improved sanitation facilities**	274 (90.7%)	135 (89.4%)
**Cooking Fuel**		
Gas	261 (86.4%)	126 (83.4%)
Wood/coal/animal dung	41 (13.6%)	25 (16.6%)
**Location of child school**		
Otherwise	153 (56.0%)	70 (49.6%)
Near factory/traffic fumes/public oven	120 (44.0%)	71 (50.4%)
N	273	141
**Proper ventilation in house**	274 (90.7%)	140 (92.7%)
**Current smoker in household**	70 (23.2%)	22 (14.6%)
**Pets in household**	74 (24.5%)	32 (21.2%)
**Wheeze at presentation**	23 (7.6%)	12 (7.9%)
**Under-weight**	110 (36.4%)	60 (39.7%)
**Stunting**	120 (40.1%)	71 (47.3%)

For peer review only

For peer review only - http://bmjopen.bmj.com/site/about/guidelines.xhtml

N	299	150
**Wasting**	51 (17.0%)	29 (19.2%)
N	300	151
**Received age adequate vaccination**	134 (44.4%)	84 (55.6%)
**History of exclusive breastfeeding**	187 (61.9%)	93 (61.6%)

Katcha housing: made up of mud, straw, wood and dry leaves

Pakka housing: rooves, walls and floors made of high quality material

Mixed: a combination of pakka and katcha

For peer review only

Table 2: Matched analysis showing effect of predictors on recurrent fast breathing

Predictors	Unadjusted		Adjusted	
	Matched OR (95% CI)	p-value	Matched OR (95% CI)	p-value
**Randomization group**				
Amoxicillin	1.29(0.87-1.91)	0.205	--	--
Placebo	Ref.		Ref.	
**Child sex**				
Male	Ref.		Ref.	
Female	1.01(0.69-1.50)	0.947	--	--
**Sibling number**				
<=3	Ref.		Ref.	
>3	1.03(0.67-1.59)	0.883	--	--
**Room sharing number**				
<=4	Ref.		Ref.	
>4	1.15(0.78-1.70)	0.469	--	--
**Housing Type**				
Katcha	2.80(1.22-6.44)	0.015	2.43(1.02-5.80)	0.045
Mixed	2.53(1.18-5.46)	0.018	2.44(1.11-5.38)	0.026
Pakka	Ref.		Ref.	
**Unimproved drinking water**	1.86(0.99-3.49)	0.053	1.72(0.89-3.36)	0.109
**Unimproved sanitation facilities**	1.16(0.61-2.21)	0.655	--	--
**Cooking Fuel**				
Gas	Ref.		Ref.	
Wood/coal/animal dung	1.28(0.73-2.22)	0.387	--	--
**Location of child school**				
Otherwise	Ref.			
Near factory/traffic fumes/public oven	1.28(0.84-1.95)	0.253	--	--
**No proper ventilation in house**	0.78(0.38-1.59)	0.487	--	--
**Current smoker in household**	0.57(0.34-0.96)	0.033	0.53(0.30-0.91)	0.022
**Pets in household**	0.83(0.52-1.33)	0.433	--	--

For peer review only

**Wheeze at presentation**	1.05(0.50-2.18)	0.901	--	--
**Underweight**	1.16(0.77-1.74)	0.486	--	--
**Stunted**	1.39(0.91-2.14)	0.129	--	--
**Wasted**	1.16(0.70-1.94)	0.566	--	--
**Age adequate vaccination**				
No	0.62(0.41-0.93)	0.021	0.61(0.40-0.94)	0.025
Yes	Ref.		Ref.	
**History of exclusive breastfeeding**				
Yes	Ref.		Ref.	
No	1.01(0.68-1.52)	0.946	--	--

For peer review only

For peer review only - http://bmjopen.bmj.com/site/about/guidelines.xhtml

Discussion

Our findings, to our knowledge are the first to show the burden of recurrence of fast breathing pneumonia and the association with poor housing quality in this setting. A total of 8.3 % of the children lived in ‘katcha’ or mixed housing, forms of mud brick buildings often made as temporary homes for the poorest families which, even by urban slum standards, are rudimentary

In the multivariable analysis poor housing quality was independently associated with recurrence with aMORs (95% CI) relative to the pukka housing reference of 2.43 (1.02- 5.80, p 0.04) and mixed 2.44 (1.11- 5.38, p 0.03) for katcha and mixed housing respectively. There was no association with: undernutrition; sex; inadequate ventilation; absence of breastfeeding; type of household fuel used; sibling number; bedroom crowding; wheeze at first presentation; household pet animals; sanitation or the proximity of the child’s school to factories, traffic fumes or public ovens.

We feel the study has a number of strengths. Firstly, though there is some published work from Low and Middle Income Countries related to environment and respiratory health, there has been little data from Asia.

Secondly, as a result of the existing infrastructure in our population, follow up was robust and capture of recurrence of pneumonia was excellent.

Thirdly, the matched design removes potential confounding effects of age and immunization status. Infants have higher incidence rates of pneumonia than older children and, for logistical reasons are more likely not to have completed routine vaccination. Matching is likely to have improved the precision of the estimate.

The study and inferences have some limitations. Firstly, the population is relatively homogenous in terms of socio-economic status which may have blunted estimated effect sizes of some potential exposures. For example, all households were poor in comparison to middle class inner city dwellers and some degree of indoor air pollution exposure was the norm. Crude nutritional status by weight for age z-score was also prevalent (underweight 36 .4 % and stunted 40%) that any differences that were might have been falsely negative. Secondly, there was lack of database linkage due to incorrectly recorded demographic surveillance identity in a small number (17.7 %) of cases. Thirdly, it is also possible that a small

For peer review only - http://bmjopen.bmj.com/site/about/guidelines.xhtml

proportion of recurrent cases may have presented to other facilities (private or out of the city) which may have led to an underestimate of incidence of recurrence. Fourth, we did not directly measure particulate matter density to quantify solid fuel exposure but used a questionnaire which might have been too insensitive to detect differences. Finally, we have inferred that housing quality is a valid proxy for poverty. While we feel this is appropriate, it might be a marker of other unmeasured exposures such as migration or recent displacement from permanent homes

Childhood pneumonia is intimately linked to poverty in all its manifestations. These include overcrowding, undernutrition, incomplete vaccination and the use of indoor air pollution (11,12, 17). Sonego's meta-analysis estimated effect sizes for a range of socio-economic predictors and found that early pneumonia mortality was significantly related to young maternal age, low maternal education and low socio-economic status (12).

Poverty is a sensitive, marker for adverse environmental stress (1-8, 10-14, 16). Not all poverty phenotypes are as easily measurable as, for example, the 'classic' markers: nutritional status; household size; completeness of vaccination; exposure to contaminated drinking water and airborne fine particulate matter. There is substantial evidence that one pathway is of chronic low grade inflammation demonstrable, for example by raised mean C reactive protein in more deprived populations (22) and of subtle micronutrient deficiencies and low level intergenerational psychological stress (23) all of which can be immune suppressant. Other factors increasing susceptibility include the mediating effects of low maternal education, maternal depression and impaired mother child interaction on childhood illness (24)

Recent Global Burden of Disease analysis indicated that poverty was the single largest contributor to childhood illness in Sub Saharan Africa (25) though any association between poverty, education, family size, sanitation and fuel use is complex. In recent work in Pakistan, Nasir showed multiple inter-related pathways (26). In Ram's study in Bangladesh in children aged under 5 years with pneumonia where comparable proportions to our study lived in households using gas (85 % vs 86 % in Karachi) suggested that improved ventilation and the reduction of overcrowding were the single largest preventable determinants of illness. Tin

For peer review only - http://bmjopen.bmj.com/site/about/guidelines.xhtml

roofs, low socio economic status and male sex were other predictors, but, as in our study particulate matter per se was not (27) .

The relative contribution of each airborne pollutant is arguably harder to ascertain epidemiologically as each of the factors, black carbon, fossil fuel, fine particulate matter (PM 2.5) and carbon monoxide (CO) , is so closely linked to all the others (7) Extrapolating this, Adaji's meta-analysis showed no association between home CO exposure and pneumonia. Fine particulate matter was only significant when using fuel as a proxy for exposure and not when measured directly highlighting the issues with standardizing exposure measurement in such settings (16)

It would be consistent with this hypothesis that household size is too blunt a tool, the majority of the families consisting of 10 or more members and multiple room sharing inevitable. Incomplete vaccination status similarly was so common (55.6 %) that interpretation of the result difficult and likely to be explained by confounding. In addition, most episodes of fast breathing pneumonia are known to be viral against which the common capsulated polysaccharide antigens are unlikely to be protective. Given the association of poverty with recurrent pneumonia and the cost of cigarettes it seems probable that the effect of household smoking is confounded by wealth. Consistent with this assertion is the association between household smoking and household wealth.

There are potential policy implications of these findings. Pneumonia is a common childhood illness and higher incidence rates likely to be a marker of general poor health. Though the associations between environmental exposures and poor WASH status are well recognized, we found an independent association with poor housing. Katcha and mixed housing are inextricably bound to poverty and might also mirror subtle nutritional deficiencies and family displacement. It is possible that Provision of higher quality housing might ameliorate this effect. There is also a case for enhanced routine health surveillance in these families. This would not be without complexity as poorer families are less likely to present to health facilities and door to door monitoring might be required

For peer review only - http://bmjopen.bmj.com/site/about/guidelines.xhtml

Conclusion

Recurrent fast breathing pneumonia in this deprived peri urban population in Pakistan was significantly independently associated with poor housing quality. It is likely that rudimentary homes are a marker of poverty. Factors other than recognised environmental exposures but inherent to poverty are likely to be involved.

Acknowledgements

We would like to acknowledge patients and families, the RETAPP study team, including community health workers, demographic surveillance workers and the committed physicians who took care of patients.

Funding

This work is jointly funded by the MRC-Wellcome-DFID through the Joint Global Health Trials, [grant number MR/L004283/1] and the Bill and Melinda Gates Foundation [grant number OPP1158281].

Fyezah Jehan and Imran Nisar received funding from the National Institute of Health's Fogarty International Center [Grant Number 1 D43 TW007585-01].

Competing interests

The authors declare no competing interests.

Authors' contributions

FJ conceived the idea; FJ, SK, IN, BB and NB designed the study. FJ, IN, BB, NB, NR were involved in conduct of the study. NB, AR, FJ, SK, IN, NB and NR were involved in statistical analysis. FJ, NB and SK wrote the first draft of the manuscript and NB rewrote new drafts based on input from co-authors IN, NB, BB and NR. All authors read and approved the final manuscript.

Ethics

The RETAPP study received ethical approval by the Ethics Review Committee of Aga Khan University (ERC reference number 2786-Ped-ERC-13).

For peer review only - http://bmjopen.bmj.com/site/about/guidelines.xhtml

Data sharing

Data will available on public domain 2 years after the completion of RETAPP trial and publication of the main manuscripts. Processes will be developed to facilitate data sharing for scientific utilization in a collaborative manner.

Figure 1. Study flow chart

Supplementary file 1: STROBE checklist

Supplementary file 2: recurrence by season

For peer review only

For peer review only - http://bmjopen.bmj.com/site/about/guidelines.xhtml

References

1. Boloursaz MR, Lotfian F, Aghahosseini F, Cheraghvandi A, Khalilzadeh S, Farjah A, et al. Epidemiology of Lower Respiratory Tract Infections in Children. J Compr Ped. 2013;3(3):93–8.

2. Levels and Trends in Child Mortality: Report 2015: Estimates Developed by the UN Inter-Agency Group for Child Mortality Estimation (IGME). World Health Organization: New York: United Nations Children's Fund.

3. Walker CLF, Rudan I, Liu L, et al. Global burden of childhood pneumonia and diarrhoea. The Lancet 2013;**381**(9875):1405-16.

4. Liu L, Oza S, Hogan D, et al. Global, regional, and national causes of under-5 mortality in 2000–15: an updated systematic analysis with implications for the Sustainable Development Goals. The Lancet 2016;**388**(10063):3027-35.

5. UNICEF 2018. https://data.unicef.org/topic/child-health/pneumonia/. Last accessed 5/10/19

6. Leung, Christi et al (2016). Prevention and Control of Childhood Pneumonia and Diarrhea. Pediatr Clin North Am. ; 63(1): 67–79. doi:10.1016/j.pcl.2015.08.003

‘

7. Rudan I, O'Brien KL et al 2013. Epidemiology and etiology of childhood pneumonia in 2010: estimates of incidence, severe morbidity, mortality, underlying risk factors and causative pathogens for 192 countries. J Glob Health. 2013;3:010401. Medline:23826505

8. Zhang S, Sammon P. Cost of management of severe pneumonia in young children: systematic analysis. Journal of Global Health 2016. doi: 10.7189/jogh.06.010408

For peer review only

9. Tuti T, Agweyu A, Mwaniki P, Peek N, English M, on behalf of the Clinical Information Network Author Group. An exploration of mortality risk factors in non-severe pneumonia in children using clinical data from Kenya. BMC Medicine. 2017 Nov 13; 15: 201

10. Scott JA, Brooks WA et al. Pneumonia research to reduce childhood mortality in the developing world. J Clin Invest. 2008;118(4):1291-1300. https://doi.org/10.1172/JCI33947

11. Niessen L, ten Hove A. Comparative impact assessment of child pneumonia interventions. Bull World Health Organ 2009;87:472–480 | doi:10.2471/BLT.08.050872

12. Sonego M, Pellegrin MC, Becker G, Lazzerini M (2015) Risk Factors for Mortality from Acute Lower Respiratory Infections (ALRI) in Children under Five Years of Age in Low and Middle-Income Countries: A Systematic Review and Meta-Analysis of Observational Studies. PLOS ONE 10(1): e0116380.https://doi.org/10.1371/journal.pone.0116380

13. Adaji EE, Phaley R, Clifford M. Understanding the effect of indoor air pollution on pneumonia in children under 5 in low- and middle-income countries: a systematic review of evidence. Environ Sci Pollut Res Int. 2019; 26(4): 3208–3225. doi: 10.1007/s11356-018-3769-1

14. Bradley RH, Putnick DL. Housing Quality and Access to Material and Learning Resources Within the Home Environment in Developing Countries. Child Dev. 2012 Jan; 83(1): 76–91. doi: 10.1111/j.1467-8624.2011.01674.x

15. Adaji EE, Ekezie W, Clifford M› Phalkey R. Understanding the effect of indoor air pollution on pneumonia in children under 5 in low- and middle-income countries: a

For peer review only

systematic review of evidence. Environmental Science and Pollution Research (2019) 26:3208–3225 https://doi.org/10.1007/s11356-018-3769-1

16. Bouazza N, Foissac F et al. Fine particulate pollution and asthma exacerbations. Arch Dis Child 2017;0:1–4. doi:10.1136/archdischild-2017-312826

17. Been JV, Sheikh A. Tobacco control policies in relation to child health and perinatal health outcomes. Arch Dis Child 2018;103:817–819.

18. World Health Organisation. https://www.ncbi.nlm.nih.gov/books/NBK264164/

19. Jehan F, Nisar MI, Kerai S, et al. A double blind community-based randomized trial of amoxicillin versus placebo for fast breathing pneumonia in children aged 2-59 months in Karachi, Pakistan (RETAPP). BMC infectious diseases 2016;**16**(1):1.

20. Jehan F, Nisar I, Kerai S, **Brown N**, Ambler G, Zaidi A. Should fast breathing pneumonia cases be treated with antibiotics? The scientific rationale for revisiting management in Low and Middle Income Countries International Journal of Infectious Diseases. International Journal of Infectious Diseases 85 (2019) 64–66. https://doi.org/10.1016/j.ijid.2019.05.035

21. Izadnegahdar R, Fox MP, Thea DM, et al. Frequency and trajectory of abnormalities in respiratory rate, temperature and oxygen saturation in severe pneumonia in children. The Pediatric infectious disease journal 2012;**31**(8):863.

22. Schmeer KK, Yoon A. Socioeconomic status inequalities in low-grade inflammation during childhood. Archives of Disease in Childhood 2016;**101:**1043-1047.

23. Burris HH, Lorch SA, Kirpalani H, et al

For peer review only - http://bmjopen.bmj.com/site/about/guidelines.xhtml

Racial disparities in preterm birth in USA: a biosensor of physical and social environmental exposures. Archives of Disease in Childhood 2019;**104:**931-935.

24. Brown N, Finch Jenna, Jelena Obradovic, Aisha Yousafzai. Maternal Care mediates the effects of nutrition and responsive stimulation interventions on young children's growth. Child: health, care and development. DOI:10.1111/cch.12466

25. Lim SS, Vos T, Flaxman AD, et al. A comparative risk assessment of burden of disease and injury attributable to 67 risk factors and risk factor clusters in 21 regions, 1990–2010: a systematic analysis for the Global Burden of Disease Study 2010. The lancet 2013;**380**(9859):2224-60.

26. Nasir ZA, Murtaza F, Colbeck I. Role of poverty in fuel choice and exposure to indoor air pollution in Pakistan. Journal of Integrative Environmental Sciences 2015;**12**(2):107-17.

27. Ram PK, Dutt D, Silk BJ, et al. Household air quality risk factors associated with childhood pneumonia in urban dhaka, bangladesh. The American journal of tropical medicine and hygiene 2014;**90**(5):968-75.

For peer review only

For peer review only

For peer review only

For peer review only - http://bmjopen.bmj.com/site/about/guidelines.xhtml

Figure I: flow chart

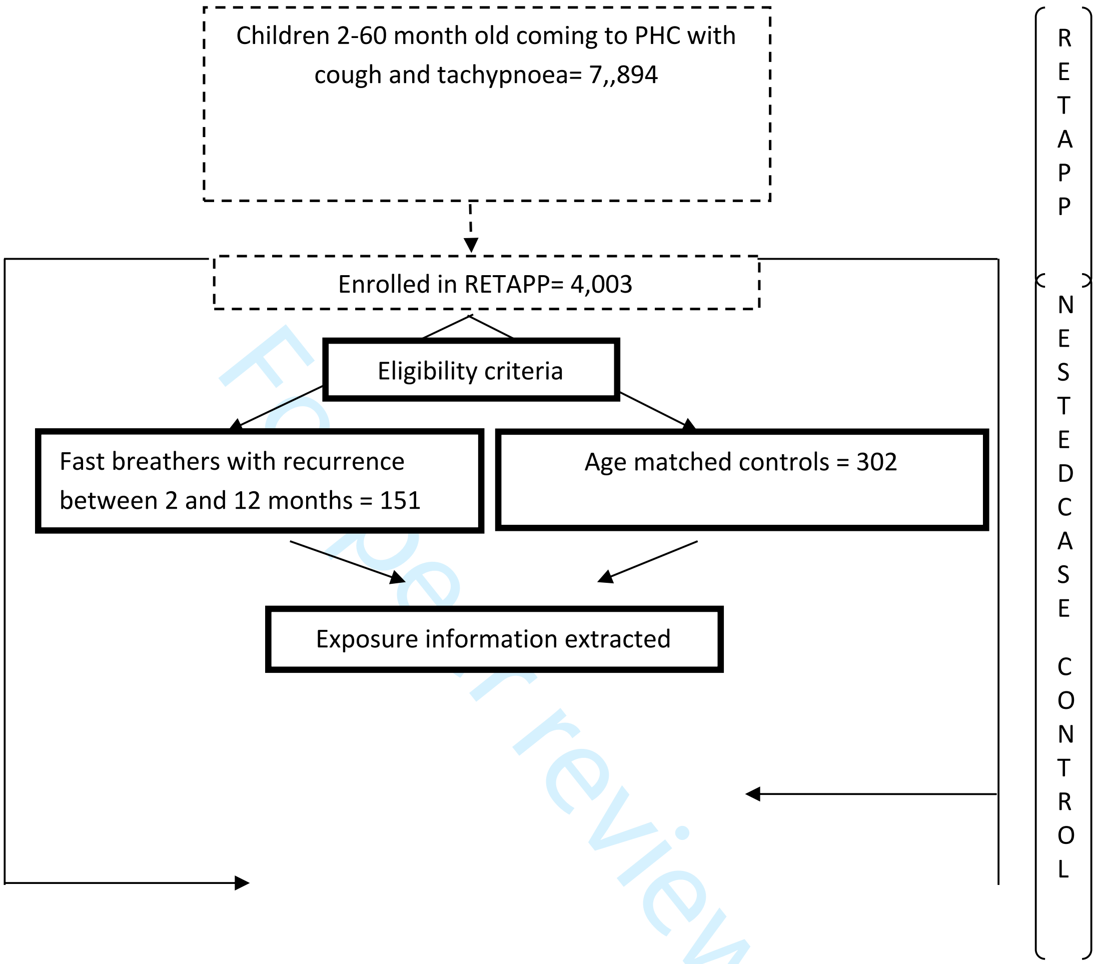

For peer review only

STROBE Statement—checklist of items that should be included in reports of observational studies

	Item No	Recommendation	Page No
**Title and abstract**	1	(*a*) Indicate the study's design with a commonly used term in the title or the abstract	Yes
		(*b*) Provide in the abstract an informative and balanced summary of what was done and what was found	Yes
**Introduction**			
Background/rationale	2	Explain the scientific background and rationale for the investigation being reported	Yes 2-3
Objectives	3	State specific objectives, including any prespecified hypotheses	2-3
**Methods**			
Study design	4	Present key elements of study design early in the paper	5-7
Setting	5	Describe the setting, locations, and relevant dates, including periods of recruitment, exposure, follow-up, and data collection	5-7
Participants	6	(*a*) *Cohort study*—Give the eligibility criteria, and the sources and methods of selection of participants. Describe methods of follow-up *Case-control study*—Give the eligibility criteria, and the sources and methods of case ascertainment and control selection. Give the rationale for the choice of cases and controls *Cross-sectional study*—Give the eligibility criteria, and the sources and methods of selection of participants	5-7
		(*b*) *Cohort study*—For matched studies, give matching criteria and number of exposed and unexposed *Case-control study*—For matched studies, give matching criteria and the number of controls per case	
Variables	7	Clearly define all outcomes, exposures, predictors, potential confounders, and effect modifiers. Give diagnostic criteria, if applicable	5-7
Data sources/ measurement	8*	For each variable of interest, give sources of data and details of methods of assessment (measurement). Describe comparability of assessment methods if there is more than one group	*5-7*
Bias	9	Describe any efforts to address potential sources of bias	5-7
Study size	10	Explain how the study size was arrived at	5-7
Quantitative variables	11	Explain how quantitative variables were handled in the analyses. If applicable, describe which groupings were chosen and why	5-7
Statistical methods	12	(*a*) Describe all statistical methods, including those used to control for confounding	5-7
		(*b*) Describe any methods used to examine subgroups and interactions	5-7
		(*c*) Explain how missing data were addressed	5-7
		(*d*) *Cohort study*—If applicable, explain how loss to follow-up was addressed *Case-control study*—If applicable, explain how matching of cases and controls was addressed *Cross-sectional study*—If applicable, describe analytical methods taking account of sampling strategy	5-7
		(*e*) Describe any sensitivity analyses	5-7

Continued on next page

For peer review only - http://bmjopen.bmj.com/site/about/guidelines.xhtml

Results			
Participants	13*	(a) Report numbers of individuals at each stage of study—eg numbers potentially eligible, examined for eligibility, confirmed eligible, included in the study, completing follow-up, and analysed	8+figure
		(b) Give reasons for non-participation at each stage	8
		(c) Consider use of a flow diagram	figure
Descriptive data	14*	(a) Give characteristics of study participants (eg demographic, clinical, social) and information on exposures and potential confounders	8+table 1
		(b) Indicate number of participants with missing data for each variable of interest	8
		(c) *Cohort study*—Summarise follow-up time (eg, average and total amount)	
Outcome data	15*	*Cohort study*—Report numbers of outcome events or summary measures over time	
		Case-control study—Report numbers in each exposure category, or summary measures of exposure	*8*
		Cross-sectional study—Report numbers of outcome events or summary measures	
Main results	16	(*a*) Give unadjusted estimates and, if applicable, confounder-adjusted estimates and their precision (eg, 95% confidence interval). Make clear which confounders were adjusted for and why they were included	8+table 2
		(*b*) Report category boundaries when continuous variables were categorized	8+table 2
		(*c*) If relevant, consider translating estimates of relative risk into absolute risk for a meaningful time period	-
Other analyses	17	Report other analyses done—eg analyses of subgroups and interactions, and sensitivity analyses	7
**Discussion**			
Key results	18	Summarise key results with reference to study objectives	13-15
Limitations	19	Discuss limitations of the study, taking into account sources of potential bias or imprecision. Discuss both direction and magnitude of any potential bias	13-15
Interpretation	20	Give a cautious overall interpretation of results considering objectives, limitations, multiplicity of analyses, results from similar studies, and other relevant evidence	13-15
Generalisability	21	Discuss the generalisability (external validity) of the study results	13-15
**Other information**			
Funding	22	Give the source of funding and the role of the funders for the present study and, if applicable, for the original study on which the present article is based	15

*Give information separately for cases and controls in case-control studies and, if applicable, for exposed and unexposed groups in cohort and cross-sectional studies.

Note: An Explanation and Elaboration article discusses each checklist item and gives methodological background and published examples of transparent reporting. The STROBE checklist is best used in conjunction with this article (freely available on the Web sites of PLoS Medicine at http://www.plosmedicine.org/, Annals of Internal Medicine at http://www.annals.org/, and Epidemiology at http://www.epidem.com/). Information on the STROBE Initiative is available at www.strobe-statement.org.

For peer review only

Supplementary file

Recurrence by season

	Recurrent Fast breathing		
	Count	%	N
Jan	15	3%	445
Feb	16	6%	281
Mar	14	4%	328
Apr	11	3%	380
May	12	3%	343
Jun	16	5%	319
Jul	12	3%	380
Aug	14	5%	297
Sep	9	3%	263
Oct	13	3%	374
Nov	8	3%	272
Dec	11	3%	320

For peer review only

Selected vs all controls

		Potential controls	Selected controls	p-value
		N=2,455	N=302	
Arm	A	1,223 (49.8%)	149 (49.3%)	0.88
	B	1,232 (50.2%)	153 (50.7%)	
Child sex	Male	1,252 (51.0%)	167 (55.3%)	0.16
	Female	1,203 (49.0%)	135 (44.7%)	
Sibling number	<=3	1,731 (70.5%)	218 (72.2%)	0.55
	>3	724 (29.5%)	84 (27.8%)	
Room sharing number	<=4	1,398 (56.9%)	173 (57.3%)	0.91
	>4	1,057 (43.1%)	129 (42.7%)	
Housing Type	Katcha	138 (5.6%)	10 (3.3%)	0.013
	Mixed	218 (8.9%)	15 (5.0%)	
	Pakka	2,099 (85.5%)	277 (91.7%)	
Location of child school	Other	1,216 (55.0%)	153 (56.0%)	0.75
	Near factory/traffic fumes/public oven	993 (45.0%)	120 (44.0%)	
Drinking water	Improved drinking Water	2,218 (90.3%)	274 (90.7%)	0.83
	Unimproved drinking water	237 (9.7%)	28 (9.3%)	
Sanitation facilities	Improved sanitation facilities	2,227 (90.7%)	274 (90.7%)	0.99
	Unimproved sanitation facilities	228 (9.3%)	28 (9.3%)	
Cooking Fuel	Gas	2,098 (85.5%)	261 (86.4%)	0.65
	Wood/coal/animal dung	357 (14.5%)	41 (13.6%)	
Proper ventilation	Yes	2,209 (90.0%)	274 (90.7%)	0.68
	No	246 (10.0%)	28 (9.3%)	
Smoker in household	Yes	656 (26.7%)	70 (23.2%)	0.19
	No	1,799 (73.3%)	232 (76.8%)	
Pets with fur/feathers in household	Yes	535 (21.8%)	74 (24.5%)	0.28
	No	1,920 (78.2%)	228 (75.5%)	
Wheeze at presentstion	Yes	146 (5.9%)	23 (7.6%)	0.25
	No	2,309 (94.1%)	279 (92.4%)	
Weight-for-age z-score	Normal	1,414 (57.8%)	192 (63.6%)	0.053
	Under-weight	1,034 (42.2%)	110 (36.4%)	
Height-for-age z-score	Normal	1,323 (54.2%)	179 (59.9%)	0.065
	Stunting	1,116 (45.8%)	120 (40.1%)	
	Normal	1,969 (81.1%)	249 (83.0%)	0.42

For peer review only - http://bmjopen.bmj.com/site/about/guidelines.xhtml

Weight-for-height z-score	Wasting	459 (18.9%)	51 (17.0%)	
Age adequate vaccination	No	1,321 (53.8%)	168 (55.6%)	0.55
	Yes	1,134 (46.2%)	134 (44.4%)	
History of exclusive breastfeeding	Yes	1,563 (63.7%)	187 (61.9%)	0.55
	No	892 (36.3%)	115 (38.1%)	

For peer review only
